# Duration of recovery from severe acute malnutrition and associated factors in children aged 6–59 months: a retrospective cohort study

**DOI:** 10.1038/s41598-025-28931-5

**Published:** 2025-12-23

**Authors:** Hikma Fedlu, Lata Fekadu, Amanuel Mengistu Merera, Kumara Asafa, Firew Tiruneh

**Affiliations:** 1 Department of Public Health Officer, Faculty of Medical Sciences, Jimma University, Jimma, Ethiopia; 2 Department of Epidemiology and Biostatistics, Faculty of Public Health, Jimma University, Jimma, Ethiopia; 3 Department of Radiology, Lancet General Hospital, Addis Ababa, Ethiopia

**Keywords:** Severe acute malnutrition, Time to recovery, Retrospective cohort, Jimma, Nutrition, Paediatrics, Public health

## Abstract

Malnutrition, particularly severe acute malnutrition (SAM) in children under five, is a global crisis affecting 25–35 million children, especially in Africa and Southeast Asia. In Ethiopia, 21% are underweight, 36% stunted, and 7% wasted. This study assesses recovery time from SAM in children admitted to public hospitals in Jimma Town. We conducted a retrospective study with 323 participants, collecting data from medical charts and analyzing it in Stata version 15. Used Kaplan-Meier for survival analysis and Cox regression for predictors of hospital stay, considering p-values < 0.05 as significant. A total of 81.1% of children with severe acute malnutrition recovered, with a recovery rate of 6.8 per 100 person-days and a median recovery time of 12 days. The predictors of nutritional recovery time were: unvaccinated (AHR = 1.630, 95% CI: 1.444, 1.894), having medical complications (AHR = 1.335, 95% CI: 1.195, 1.576), tuberculosis (AHR = 1.565, 95% CI: 1.395–2.809), pneumonia (AHR = 2.668, 95% CI: 2.405–2.769), being non-edematous (AHR = 1.880, 95% CI: 1.278, 2.765), marasmic kwashiorkor (AHR = 2.000, 95% CI: 1.254, 3.486), and hospital-acquired infections (AHR = 1.459, 95% CI: 1.315, 1.669). The study found optimal median recovery time influenced by vaccination status, comorbidities, and medical complications. Improved vaccination coverage, timely treatment, and infection control reduce hospital stays for severe acute malnutrition.

**Supplementary Information:**

The online version contains supplementary material available at 10.1038/s41598-025-28931-5.

## Introduction

Malnutrition remains one of the most pressing public health challenges globally, particularly affecting vulnerable populations such as children under five years of age^1^. Defined broadly, malnutrition encompasses both undernutrition characterized by deficiencies in energy, protein, and micronutrients and over nutrition, which leads to overweight and obesity. Among the various forms of undernutrition, severe acute malnutrition (SAM) is the most critical, as it significantly increases the risk of morbidity and mortality in young children^2–4^.

Globally, an estimated 25–35 million children suffer from severe acute malnutrition (SAM), particularly in areas with food insecurity, conflict, and poor healthcare^5^. The WHO states that children with SAM are up to 11 times more likely to die than well-nourished peers^2^. Malnutrition has long-term effects, leading to developmental deficits, reduced cognitive function, and increased chronic disease risk^6,7^. In 2021, around 149.2 million children under 5 were stunted, 45.4 million wasted, and 38.9 million overweight^5^. SAM affects nearly 20 million children under 5, mainly in Africa and South-East Asia, causing up to 1 million deaths annually due to increased infection susceptibility^8–10^.

Ethiopia has been identified as one of the countries with a high burden of malnutrition. According to recent national surveys, approximately 21% of children under five are classified as underweight, 36% were stunting and 7% suffer from wasting^11^. These statistics reflect the multifaceted nature of malnutrition in Ethiopia, which is influenced by factors such as poverty, inadequate dietary diversity, poor maternal health and nutrition, and recurrent environmental shocks such as drought and conflict^12–14^. The Ethiopian government, alongside international organizations, has implemented various programs aimed at addressing malnutrition through community-based management of acute malnutrition (CMAM), nutrition education, and food supplementation initiatives^4^.

Recent studies in Ethiopia indicate recovery rates in stabilization centers (SC) are below standards^11,12^, while others align with these standards^13–15^. A similar study in Uganda reported an unacceptably high death rate^16^. Factors such as age, breastfeeding, immunization, and co-morbidities at admission influenced recovery times and SC outcomes^11,14,15^. Co-morbidities like diarrhea, pneumonia, HIV status, and anemia were independent predictors of treatment outcomes^17–19^. Additionally, variables including type of severe acute malnutrition (SAM), intravenous fluid resuscitation, nasogastric feeding, appetite, and admission status significantly affected therapy outcomes in SC^13,16,17,20^.

Despite these efforts, challenges persist in treating children with SAM in inpatient settings. Research highlights that age, sex, vaccination status, co-existing infections, and complications during hospitalization significantly impact recovery^15,21–24^. However, much existing literature focuses on outpatient protocols and community interventions, leaving a gap in understanding inpatient care dynamics for severely malnourished children.

Moreover, there are variability of predictors and rate of recovery shown by different studies conducted before^18,19,25^ and in Ethiopia there is a scarcity of hospital-based studies done to explicit predictors of recovery from malnutrition among 6–59 months children. This study aims to fill a research gap by investigating the factors affecting hospital stays and recovery outcomes for children with SAM in public hospitals in Jimma, Ethiopia. By identifying key determinants of inpatient treatment efficacy, the research seeks to provide insights that can enhance clinical practices and policies to improve recovery rates and reduce mortality in this vulnerable population. Ultimately, better understanding inpatient care for malnutrition will aid efforts to combat child malnutrition and improve health outcomes.

### Significance of the study

This study is significant as it addresses a critical gap in the understanding of inpatient care for children aged 6–59 months suffering from SAM in Ethiopia, where the majority of existing literature focused on outpatient protocols and community interventions. By examining the factors influencing recovery times and hospital stays in public hospitals, this study aims to identify key determinants that affect treatment outcomes. This study will offer important insights into the effectiveness of inpatient treatment for SAM, ultimately leading to better clinical practices and policy, given the variability of recovery rates and predictors revealed in previous studies. The findings are expected to inform strategies that enhance recovery rates and reduce mortality among this vulnerable population, thereby supporting broader efforts to combat child malnutrition and improve overall health outcomes in Ethiopia.

## Methods and materials

### Study area and period

The study took place in Jimma; a town situated 356 km from Addis Ababa, with a population of 207,573 across 17 kebeles. Located in the Oromia Region at latitude of 7°40′ N and longitude of 36°50′ E, Jimma is home to the only specialized hospital in southwestern Ethiopia, the Jimma University Medical Center, which serves a catchment area of 15 million. The town has two hospitals, four health centers, and eight health posts, staffed by 206 health workers and 274 support staff, along with 104 private facilities. This research focused on public hospitals in Jimma, including JUMC, from February to June 2023, reviewing records from Jimma University Medical Center and Shenen Gibe General Hospital for the period of January 2020 to December 2021.

### Study design

A retrospective cohort study design was used based on secondary records of children aged 6 to 59 months with SAM admitted at the Jimma medical center and Shenen Gibe General hospital during a specified study period.

### Study population

All under-five children aged 6–59 months with severe acute malnutrition who were admitted to therapeutic feeding units in public hospitals of Jimma Town between January 2020 and December 2021.

### Eligibility criteria

#### Inclusion criteria

All medical records of 6–59 months old children with a diagnosis of SAM who were managed in therapeutic feeding units at the Jimma medical center and Shenen Gibe General Hospital therapeutic feeding center from January 2020 to December 2021 were included according to the WHO’s criteria for admission to therapeutic feeding units were, children aged 6–59 months can be classified as having SAM if they have a weight-for-height measurement below − 3 standard deviations from the median WHO growth standards, a MUAC of less than 11.5 cm, visible severe wasting, or nutritional edema^26^.

#### Exclusion criteria

Children’s medical records for those aged 6 to 59 months with Severe Acute Malnutrition (SAM) who were treated in therapeutic feeding units at public hospitals in Jimma town between January 2020 to December 2021 were reviewed. However, records with incomplete information (such as unclear diagnoses, missing anthropometric measurements, and other pertinent factors including age, date of diagnosis, treatment methods, and the child’s most recent health status) were excluded from the study. Additionally, cases transferred from other institutions with an unclear date of diagnosis were also excluded.

### Sample size determinations

The determination of sample size for factors influencing treatment recovery time in cases of Severe Acute Malnutrition (SAM) was conducted using STATA version 15. Parameters were derived from previous studies conducted in various areas^15,27^ and a review of diverse literature, focusing on the most significant factor with the largest sample size, as summarized in Table 1. In most of the literature reviewed amoxicillin was found to be significantly associated with recovery. The calculations were based on a 95% confidence interval (α = 0.05), an observed recovery probability of 0.73, 80% power, hazard ratios of 1.54, an estimated number of events (E) of 214, and a two-sided test. Ultimately, the total sample size required was determined to be 293, and after accounting for a 10% rate of missing or dropout data, the final sample size was adjusted to 323.

**Table 1:** Sample size calculation by searching determinants from different related literature for the predictor of recovery time from SAM among under five children in Jimma town public hospital from January 2020 to December 2021.

Predictors	CI	Power	AHR	Event	PE	*N*	Final sample size
Play stimulation	95	80	1.93	92	0.73	126	139
Tuberculosis	95	80	0.48	71	0.73	98	108
Amoxicillin	95	80	1.54	214	0.73	293	323
Deworming	95	80	1.8	115	0.73	158	174

*CI confidence interval 95%.

*Power 80%.

*AHR adjusted hazard ratio.

*PE probability of an event.

In Jimma town, a total of 629 children with severe acute malnutrition were admitted to therapeutic feeding units at two public hospitals: Jimma Medical Center and Shenen Gibe General Hospital. Based on proportional allocation according to the size of each hospital, 453 children were from Jimma Medical Center and 176 from Shenen Gibe General Hospital. Using simple random sampling, 232 children were selected from Jimma Medical Center and 91 from Shenen Gibe General Hospital. Consequently, the final sample size for the study was 323.

### Sampling procedure

**Fig. 1:**
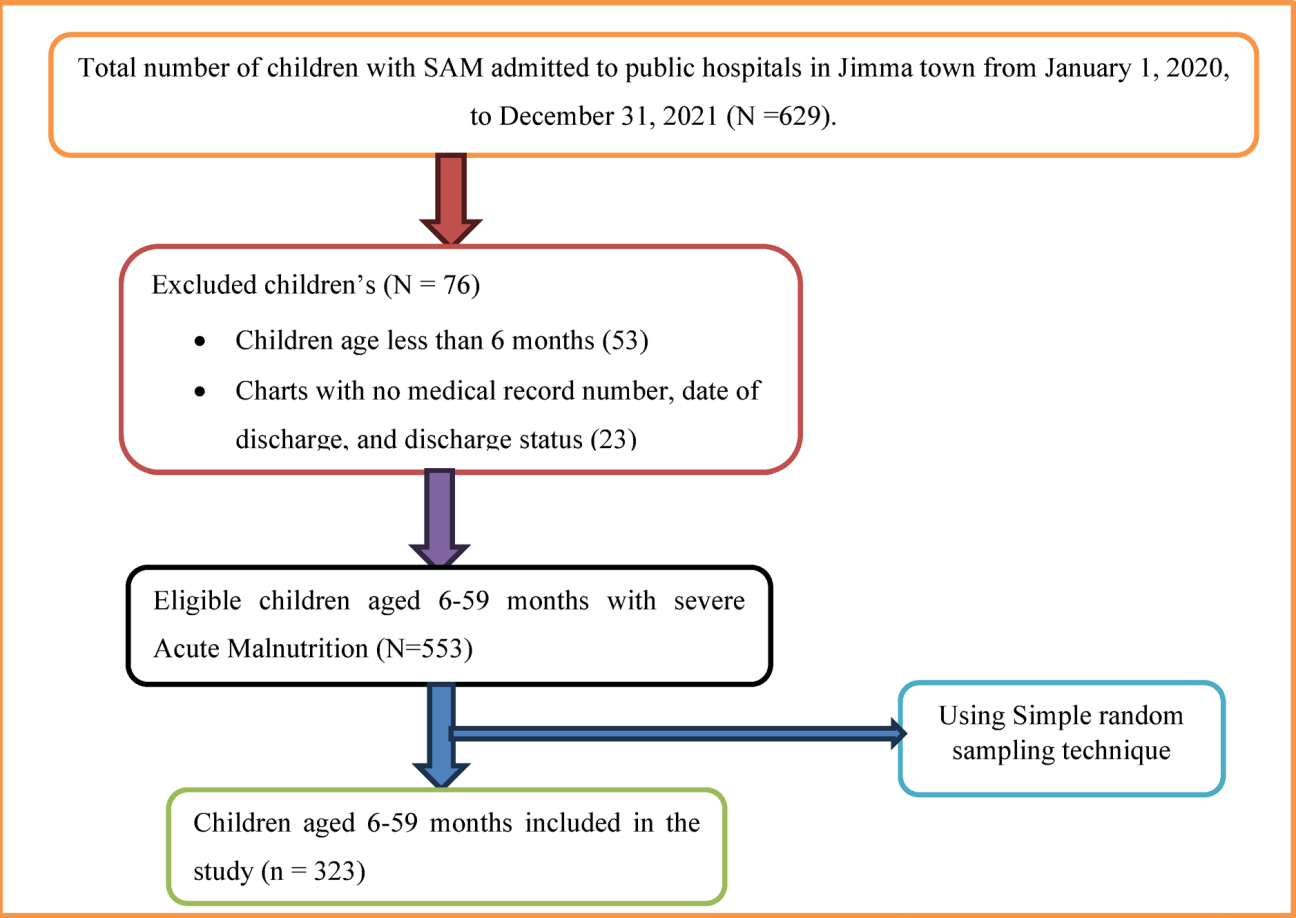
Schematic presentation of sampling procedure.

First, the total number of children with acute malnutrition was identified at each public hospital. Then the sample size was distributed to each therapeutic feeding unit using proportional allocation to size (PAS). Then the medical record number (MRN) of children who were admitted to Jimma university medical center (JUMC) and Shenen Gibe General Hospital (SGGH) Therapeutic Feeding Center from January 1, 2020 to December 31, 2021 was obtained from the registration book. Then, those records of children that do not fulfill the inclusion criteria were excluded. A computer-generated simple random sampling technique was employed to recruit a sample from the sampling frame. According to the eligibility criteria, the data was extracted from eligible medical records (See Fig. 1).

### Data collection tool and procedure

A pretested structured data abstraction tool was utilized for data collection. This tool was adapted from the Ethiopian Federal Ministry of Health’s updated SAM management guidelines from 2019^28^, along with relevant published studies. The data extraction format was created in English and translated into Amharic and Afan Oromo, adhering to census bureau guidelines for translation, ensuring semantic and normative equivalence. Data collectors underwent two days of training, and data was gathered by identifying medical record numbers from the registration book, with charts sourced from the medical records room. Charts meeting the inclusion criteria were reviewed, and necessary information was extracted using the established format. The data collection was conducted by two trained BSc pediatric nurses under supervision.

### Study variables

#### Dependent variable

Time to recovery from severe acute malnutrition.

#### Independent variables

Predictor variables for recovery time from severe acute malnutrition (SAM) include various factors: socio-demographic factors like age, sex, residence, season of admission, vaccination status, facility type, and breastfeeding. Key anthropometric measurements at admission are weight, height, and Mid-Upper Arm Circumference (MUAC) for children 6–59 months, along with weight gain and diagnoses (Marasmus, kwashiorkor). Medical co-morbidities and vital signs such as pneumonia, tuberculosis, dehydration, consciousness level, and shock. Treatment factors include IV fluid intake, antibiotics, deworming, routine medications (e.g., amoxicillin), and special medications. Nutritional therapy factors include nasogastric (NG) tube use and intake of therapeutic foods (e.g., Plumpy Nut) and milk formulas (F-100, F-75), which are predictors for recovery from SAM among aged 6–59 months children admitted to inpatient therapeutic program in Jimma town Public Hospital.

### Operational and standard definition

Recovered: When the child reaches greater or equal to 85% of median WFH or WFH Z score greater than or equal to minus 2 on more than one occasion or no edema for 10 days^29^.

Censored: Those SAM children who were defaulted, transferred, and dead were considered censored observations. Defaulters: Defined as those who are absent for two consecutive days. Death: Refers to the patient who died while he/she was in the program.

Weight change: is defined as the difference in weight between admission and discharge, which may include either gain or loss^29^.

Transfer out: referred to those who were transferred to outpatient management from inpatient management phases.

Special medication: referred to medications given to those SAM patients in addition to routine medications.

Vaccination: is defined as administering a vaccine to stimulate immunity against diseases, categorized as fully vaccinated, partially vaccinated, or unvaccinated per the Ethiopian EPI schedule.

Sunlight exposure: defined as exposure to natural sunlight for ≥30 minutes per day during admission, based on notes recorded in the nursing and nutrition follow-up charts.

Severe Acute Malnutrition: was defined as per WHO and national guidelines as: Weight-for-height Z-score < − 3 SD, or MUAC < 11.5 cm, or Presence of bilateral pitting edema.

MUAC was measured in centimeters (cm) using standard MUAC tapes and their cutoff points are, MUAC < 11.5 cm, 11.5–12.5 cm and ≥ 12.5 cm are severe acute malnutrition, moderate malnutrition and normal respectively.

Complicated and Uncomplicated Severe Acute Malnutrition (SAM): Complicated SAM refers to cases where children present with comorbidities such as pneumonia, diarrhea, tuberculosis, dehydration, or hospital-acquired infections. In contrast, Uncomplicated SAM pertains to children who do not exhibit any of these complications.

### Data quality control

To ensure data quality, a pretest was conducted on 5% of the study population at Agaro Primary Hospital to validate the data abstraction format. Errors found during this pretest were corrected, leading to a finalized version of the format. Data collectors received comprehensive training before data collection began. A data analysis mapping was also created to align the collected data with the intended analysis. Regular supervision and checks were implemented to ensure data completeness and consistency. Collected data was reviewed daily for accuracy. Data analysis plans were established before collection, and the data was analyzed every three days to monitor progress and identify any issues.

### Data processing and analysis

This study’s primary outcome was recovery time from severe acute malnutrition (SAM), with participants lost to follow-up, defaulted, died, or transferred being censored. Outcomes were classified as either censored or recovered, and data were entered using Epi Data Version 4.6 and analyzed with Stata Version 15. Descriptive statistics summarized categorical and continuous variables. Survival analysis estimated cumulative survival proportions using the Kaplan-Meier curve and log-rank test. A bivariate Cox proportional hazards regression identified candidate variables for multivariable analysis to find independent predictors of recovery time, focusing on covariates linked to hospital stay duration with a p-value of 0.25 or lower. Multicollinearity was assessed using the variance inflation factor (VIF). The multivariable Cox model calculated adjusted hazard ratios and 95% confidence intervals. A p-value under 0.05 indicated significant associations, while the proportional hazard assumption was verified using Schoenfeld residuals and Cox-Snell residuals.

### Ethics approval

The study was approved by the institutional review board of Jimma University Institute of Health (JUIH/IRB/478/23). The hospital administrators were informed that the information obtained from patient’s medical records would be kept with complete confidentiality. All participants, including minors’ parents/legal guardians and illiterate respondents, provided written informed consent to participate in the study. No personal identifiers were used during data collection to maintain anonymity and patient confidentiality. All methods and procedures in the study were performed in accordance with the Declaration of Helsinki.

## Results

### Description of characteristics of study participants

A total of 323 children with severe acute malnutrition were reviewed; of these, 12 (3.7%) cases were not included in the study because the necessary data was either missing or replaced with other medical charts in accordance with the predefined inclusion criteria. As a result, 323 patient medical records were included in the study, resulting in a 100% response rate. The average age of these children was 24.78 months, with a standard deviation of 18.09 months. Among the records reviewed, 163 (50.5%) were males and 160 (49.5%) were females. Approximately 54% of the participants lived in the Jimma woreda. The study also revealed that 80% of the children were exclusively breastfed, and 62.5% had received vaccinations appropriate for their age or were fully vaccinated (See Table 2).

**Table 2:** Socio-demographic characteristics, of under-five children admitted to public hospitals in Jimma town from January 2020 to December 2021, Jimma, Ethiopia.

Variables	Variable Categories	Number	Percentage
Age group in month	6–12	120	37.1%
	13–24	60	18.5%
	25–36	50	15.4%
	37–48	50	15.4%
	48–60	43	13.3%
Sex	Male	163	50.5%
	Female	160	49.5%
Residence	Jimma town	127	39.3%
	Woreda’s of Jimma	174	53.9%
	Outside of Jimma	22	6.8%
Exclusively breastfed	Yes	258	79.9%
	No	65	20.1%
Vaccination	Vaccinated	202	62.5%
	Partially vaccinated	35	10.8%
	Unvaccinated	86	26.6%
Sunlight exposure	Properly exposed	296	91.6%
	Not exposed	27	8.4%
Caregiver/parent/guardian	Caregivers present	310	96.0%
	No caregiver	13	4.0%

### Clinical factors

As indicated in supplementary Table S1, the study assessed the vital signs and anthropometric measurements of the children, revealing that the most frequently observed clinical features were wasting, edema, and abnormal vital signs. The findings highlighted that tachycardia in 31.9% of cases, tachypnea in 33.4%, and hypothermia in 62.6%. In terms of anthropometric data, the prevalence of stunting was 66.9%, while wasting and underweight were noted in 75% and 80% of the children, respectively. Among the participants, 62.2% presented with non-edematous malnutrition, and 25.4% with edematous malnutrition, with a significant portion (20.1%) exhibiting grade 3 edema. The study reported a new admission rate of 94.1%, with the majority of children arriving in an alert mental state (87%).

### Comorbidities and complications

In the study, children with comorbidities or clinical complications were classified as having complicated severe acute malnutrition (SAM). Among these children, 92% were diagnosed with complicated SAM, while only 8% had uncomplicated SAM. The most frequently observed comorbidities included tuberculosis (29.4%) and pneumonia (28.1%). Upon admission, the most prevalent complications reported were diarrhea (47.0%) and dehydration (33.4%). During their hospital stay, about 25% developed a hospital-acquired infection, of which 79.7% were of unspecified origin (See supplementary file of Table S1).

### Treatment related factors

Patients admitted to the stabilization centers received care according to national guidelines. Following the WHO treatment protocols, children were provided with various nutritional formulas, including F-75 (83.3%), plumpy nuts (52.3%), and F-100 (28.5%), along with vitamin A (13.6%) and folic acid (13.3%) supplements. Antibiotic therapy was administered both intravenously (69.7%) and orally (31.1%). The most frequently used intravenous antibiotics were gentamicin (81.9%) and ampicillin (55.94%), while amoxicillin (81.1%) and azithromycin (6.9%) were the primary oral antibiotics prescribed. Additionally, supplementary medications, including vitamin B6, multivitamins, vitamin D, and zinc oxide, were utilized in 14.4% of the cases (See supplementary file of Table S2).

### Median survival time

**Fig. 2:**
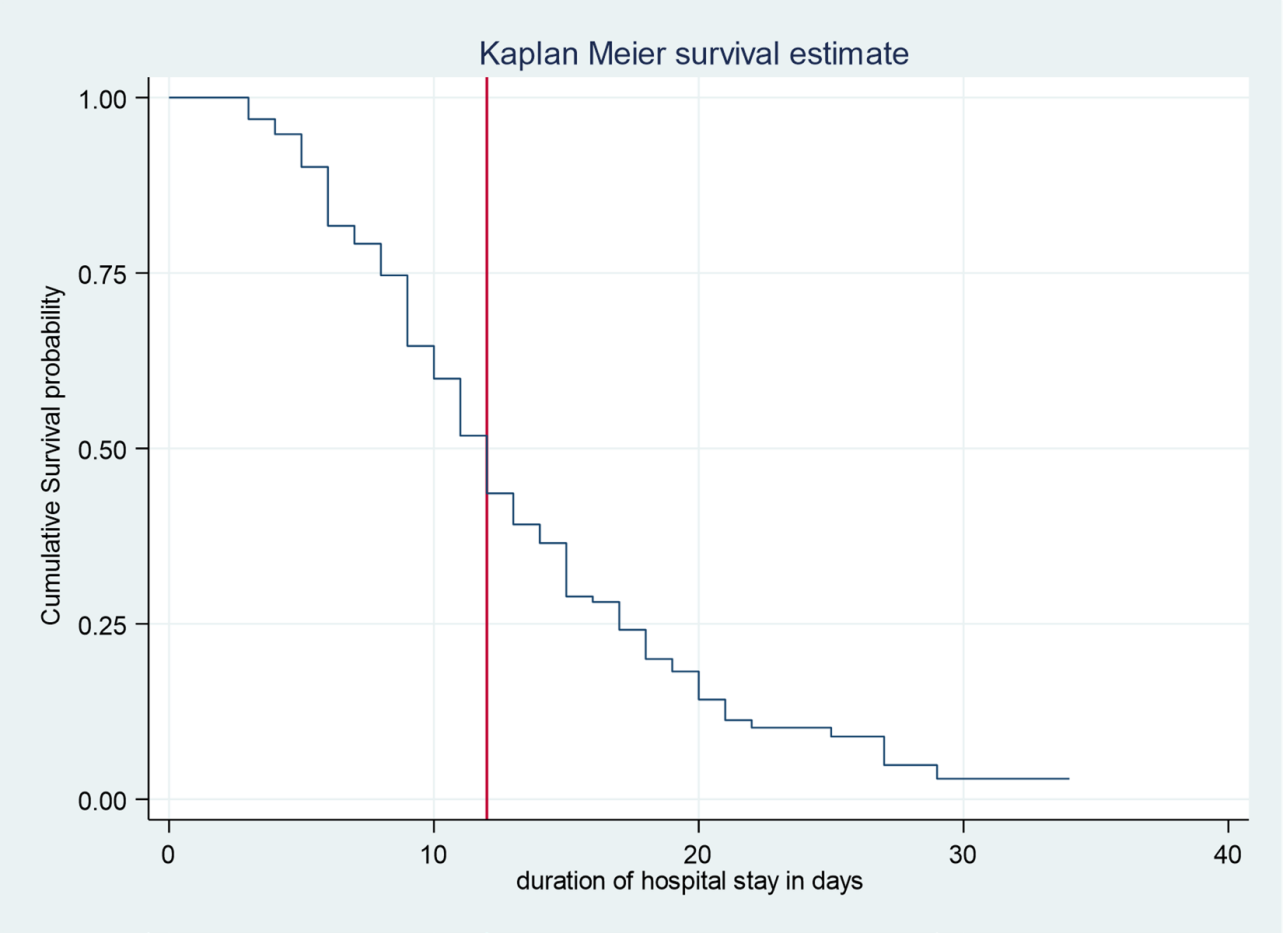
Overall Kaplan Meier Estimation for Under-5 Children with SAM Admitted to Public Hospitals in Jimma Town from January 2020 to December 2021, Jimma, Ethiopia.

A total of 323 children were monitored over a cumulative period of 3,879 person-days. Among these, 262 children achieved recovery, resulting in a recovery rate of 81.1%. The overall incidence rate was 6.8 cases per 100 child-days of observation (95% CI: 6.03–7.67). Recovery rates for children with complicated severe acute malnutrition (SAM) and uncomplicated SAM were 80.4% and 88.5%, respectively. Additional outcomes included a death rate of 10.5% and a default rate of 8.3%. The median recovery time was 12 days, with an interquartile range of 7 days. Most children experienced recovery between days 5 and 10, at a rate of 7.73 per 100 child-days of observation (See supplementary file of Figure S1, and Fig. 2).

Kaplan-Meier survival curve was used to estimate the survival status of children with SAM and indicates that most children recovered within twenty days.

### Comparison of recovery status

**Fig. 3:**
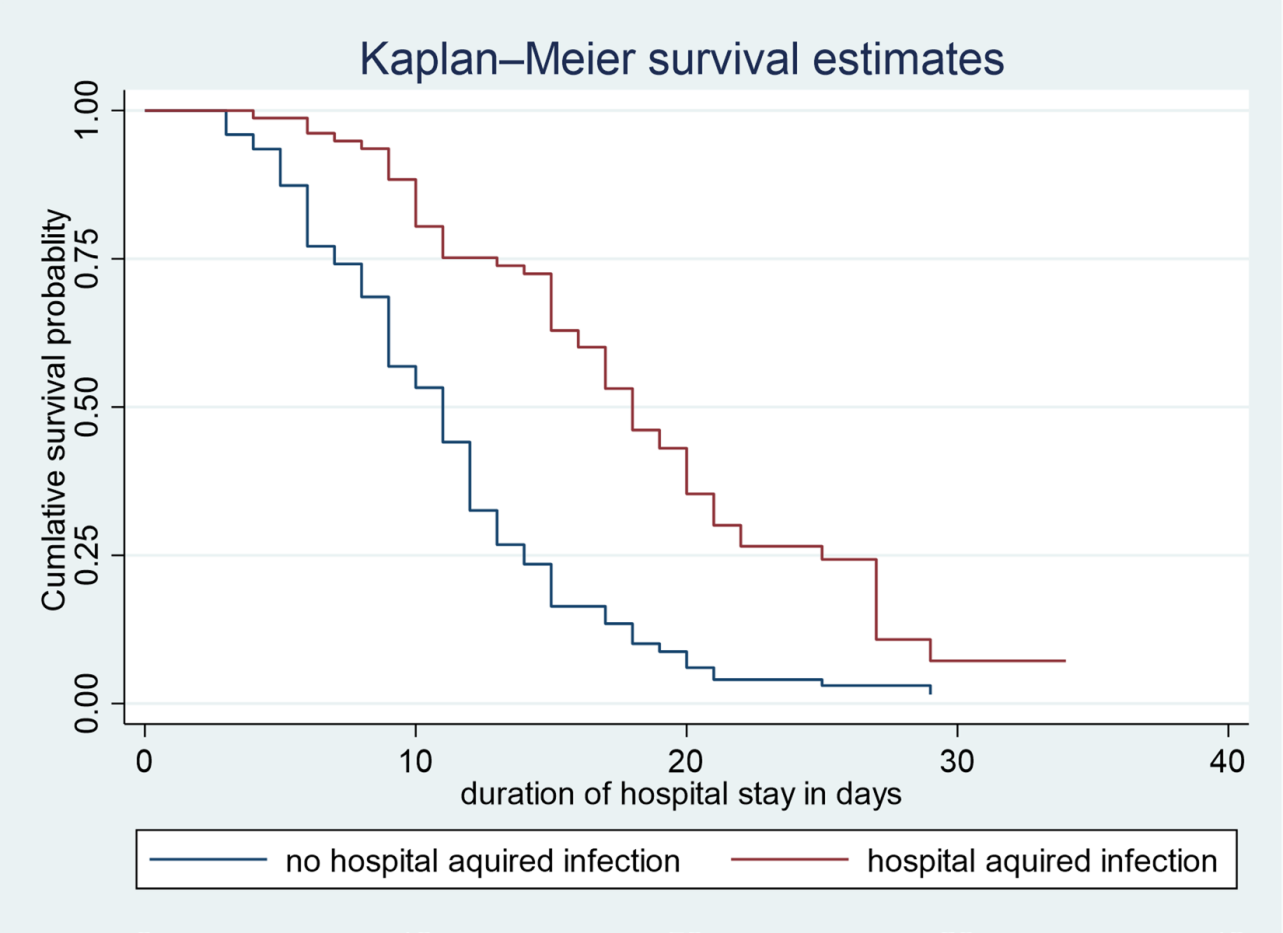
Kaplan Meier survival curve of children in public hospital in Jimma by Hospital Acquired Infections during treatment, Jimma, Ethiopia.

**Fig. 4:**
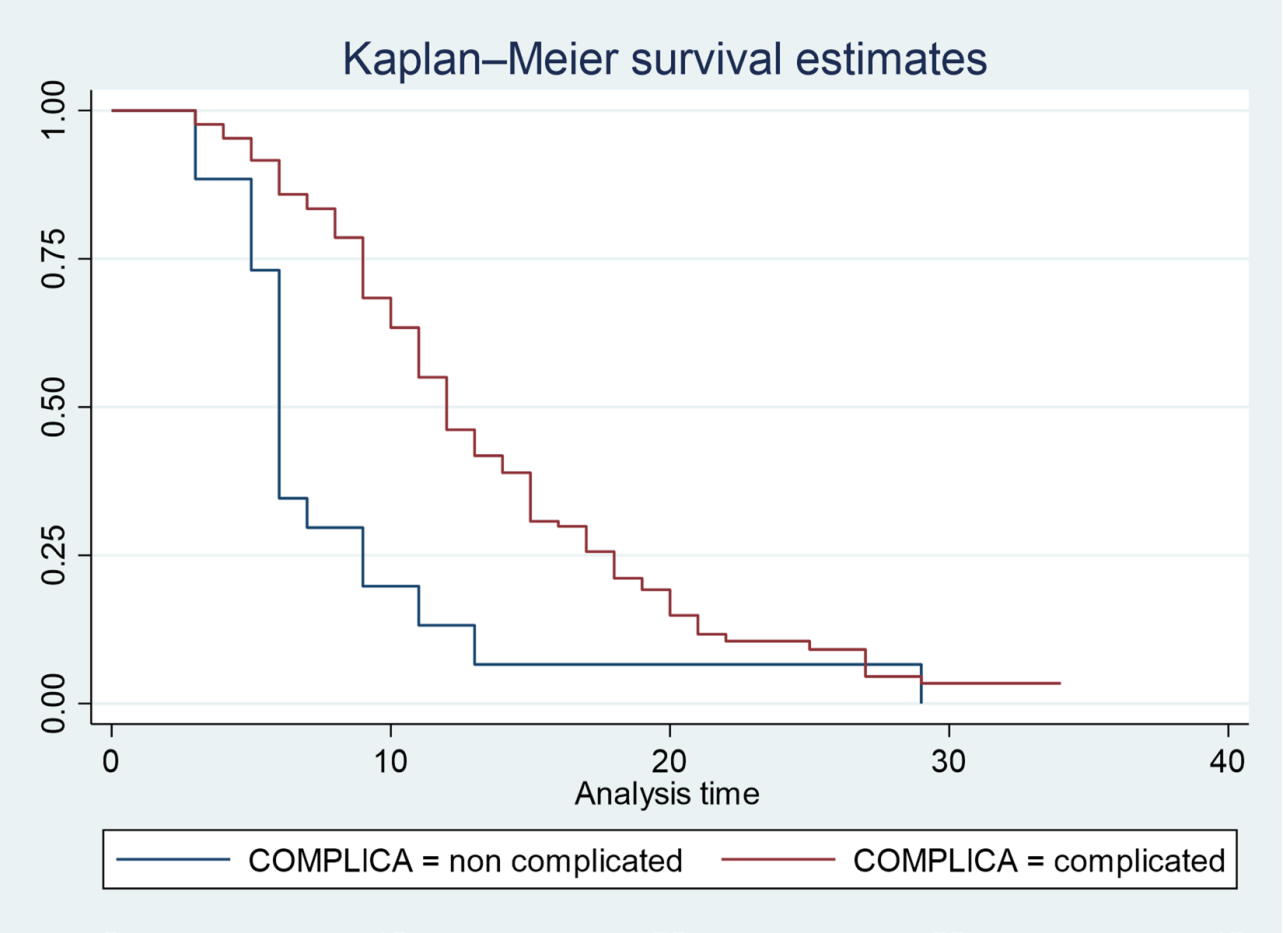
Kaplan Meier survival curve of children in public hospital in Jimma by complication status during treatment, Jimma, Ethiopia.

A Kaplan-Meier survival analysis was conducted to compare the survival probabilities across various categories of selected predictors. Additionally, the log-rank test was utilized to assess significant differences in survival probabilities among these predictors. The survival times associated with different predictors, such as complicated SAM and hospital-acquired infections (See Fig. 3, and 4), were illustrated. Notable variability in survival times was found at a 95% confidence interval for vital signs and anthropometric measurements (WFH, WFA, and HFA), as well as for conditions like TB and pneumonia, and treatments such as F-100 and F-75 (See Table 3).

**Table 3:** Log rank test and median recovery time of predictors for under five children with SAM admitted to public hospitals in Jimma Town, from January 2020 to December 2021 Jimma, Ethiopia.

Variable	Categories	Median recovery time	Incidence density	*P*-value	Chi-square Value
Pulse rate	Bradycardia	11 days: CI (9–22)	0.048	0.0003	16.37
	Normal	10 days: CI (10–12)	0.075		
	Tachycardia	14 days: CI (12–15)	0.058		
Respiratory rate	Bradypnea	9 days: CI (9–22)	0.048	0.032	6.86
	Normal	11 days: CI (10–12)	0.073		
	Tachypnea	13 days: CI (12–15)	0.0617		
WFH	Normal	11 days: CI (10–12)	0.078	0.010	9.15
	Moderate acute malnutrition	11 days: CI (9–15)	0.074		
	Severe acute malnutrition	13 days: CI (12–14)	0.062		
WFA	Normal	11 days: CI (9–12)	0.058	0.045	6.17
	Moderately underweight	12 days: CI (10–12)	0.057		
	Severely underweight	13 days: CI (12–15)	0.054		
HFA	Normal	9 days: CI (8–10)	0.091	0.00	42.79
	Moderately stunted	13 days: CI (12–15)	0.063		
	Severely stunted	15 days: CI (11–17)	0.057		
Consciousness	Alert	11 days: CI (9–12)	0.071	0.04	6.25
	Lethargic	10 days: CI (10–12)	0.057		
	Comatose	13 days: CI (12–15)	0.023		
TB	Yes	15 days: CI (15–17)	0.049	0.000	27.78
	No	10 days: CI (9–12)	0.079		
Pneumonia	Yes	17 days: CI (14–17)	0.047	0.000	36.48
	No	10 days: CI (10–11)	0.080		
HAI	Yes	18 days: CI (16–20)	0.045	0.000	49.68
	No	11 days: CI (10–12)	0.080		
F-100	Yes	15 days: CI (12–15)	0.043	0.003	8.82
	No	10 days: CI (11–12)	0.065		
F-75	Yes	15 days: CI (11–17)	0.044	0.0494	3.86
	No	12 days: CI (11–12)	0.071		

The data presented in Fig. 3 indicates that, children who did not experience any hospital-acquired infections demonstrated a significantly shorter recovery time compared to those who developed such infections during their hospital stay. This suggests that the presence of hospital-acquired infections may adversely affect the healing process, prolonging the duration of recovery for affected children. The findings highlight the importance of infection control measures in pediatric care settings to enhance recovery outcomes for vulnerable patients. Figure 4 demonstrates that, children who did not experience complications during their admission had a shorter recovery time compared to those who did have complications.

### Predictors of time to recovery from severe acute malnutrition’s

Nineteen variables that showed a P value of less than 0.25 in the bivariate analysis were included in the multivariate Cox regression analysis. Among these, the age of the children, pulse rate at admission, respiratory rate at admission, weight for age, level of consciousness at admission, malaria infection, rickets, dehydration, feeding with F-100, addition of nutritional supplements, anthropometric measurements, and deworming were determined to be insignificant in the multivariate Cox regression analysis. The significant variables are presented in Table 4 below.

As shown in Table 4, type of malnutrition at presentation had a significant effect in time to recovery. Children who presented with non-edematous malnutrition (AHR = 1.88, 95% CI: 1.278–2.765) and Marasmic Kwash (AHR = 2.00, 95% CI: 1.254–3.486) had shorter recovery time compared to children presenting with edematous malnutrition. The length of hospital stay was significantly influenced by immunization status. Unvaccinated children’s recovery time was 63% longer (AHR = 1.630, 95% CI: 1.444–1.894) when compared to children who have received the recommended vaccinations.

In general, comorbidity and complication status moreover affect how long the children remain in the hospitals in comparison to children admitted without complications and comorbidities. The study revealed that children without any complication have 33.5% better recovery than children with complications (AHR = 1.335, 95% CI: 1.195–1.576). Children with comorbidities such as tuberculosis (TB) and pneumonia experienced significantly longer recovery times compared to those without these conditions (AHR = 1.565, 95% CI: 1.395–2.809 and AHR = 2.558, 95% CI: 2.405–2.769, respectively). This indicates that children without TB and pneumonia recovered more quickly. Furthermore, children who have had a hospital-acquired infection take longer to recover 45.9% (AHR = 1.459, 95% CI: 1.315–1.669) than those who have not had a hospital-acquired infection (See Table 4).

**Table 4:** The results of the bivariable and multivariable Cox proportional hazards models among the children under five with SAM at Jimma town public Hospital, Jimma, Ethiopia, 2023 (*n* = 323).

Variables	Categories	Event to recovery		CHR (955% CI)	AHR (95% CI)	P-value
		Recovered	Censored			
Vaccination Status	Vaccinated(ref)	183	19	1	1	
	Unvaccinated	69	17	1.489(1.363–1.660)	1.630(1.444–1.894)	0.010*
	Partially Vaccinated	28	7	1.092(0.927–1.713)	1.125(1.325–1.661)	0.071
Types of Malnutrition’s	Edematous (ref)	67	15	1	1	
	Non Edematous malnutrition’s	168	33	1.099(0.826–1.462)	1.88(1.278–2.765)	0.001*
	Marasmic Kwash	33	7	1.198(0.764–1.876)	2.00(1.254–3.486)	0.014*
Children with complications	Yes (ref)	239	58	1	1	0.000*
	No	23	3	1.338(1.218–1.523)	1.335(1.195–1.576)	
Tuberculosis	No(ref)	193	35	1	1	0.000*
	Yes	69	26	1.504(1.382–1.665)	1.565(1.395–2.809)	
Pneumonia	No(ref)	197	35	1	1	0.002*
	Yes	65	26	1.447(1.336–2.595)	2.558(2.405–2.769)	
Hospital acquired infections	No(ref)	203	41	1	1	0.000*
	Yes	59	20	1.371(1.274–1.502)	1.459(1.315–1.669)	

### Test of proportional hazard assumption

The proportional hazard assumption of the regression analysis was evaluated using both statistical and graphical methods. A Cox-Snell residual plot was created to assess the overall goodness of fit of the Cox regression model. The results indicated that the Cox-Snell residuals closely followed the reference line at a 45-degree angle, confirming that the model fits the data well (see supplementary file of Figure S2). Additionally, the global test statistic for the Cox regression model was 0.22, which is greater than 0.05, indicating that the proportional hazard assumption holds true.

## Discussion

The findings of this research shed light on the duration of recovery from acute malnutrition and the various factors that affect this recovery time. The study indicates that Recovery from SAM had a median duration of 12 days. Key factors associated with the time to recovery include complicated SAM, vaccination status, comorbidities such as tuberculosis and pneumonia, the type of malnutrition, and hospital-acquired infections.

According to the international reference standards for severe acute malnutrition (SAM) indicators in feeding units, an acceptable outcome includes over 75% recovery, less than 10% mortality, and fewer than 15% defaulters, with a mean length of stay of 28 days^30^. Our study finding confirmed that, the time to recovery is optimal and align with previous research conducted in selected hospitals in Ethiopia^31^. However, the recovery time reported in our study is shorter than that observed in other studies from St. Paul Hospital in Aksum Tigray and Pawi Hospital in Benshangul Gumuz^13,25,32^. This discrepancy may reflect differences in study populations and settings as well as improvements in health care facilities.

The recovery rate and incidence density observed in this study align with the SPHERE criteria and are consistent with findings from studies conducted in Finote Selam, which reported recovery rates and incidence densities within an acceptable range^33^. In contrast, a previous study in Jimma town reported a lower incidence density^15^. This discrepancy may be due to several factors, including differences in the study populations, variations in treatment protocols, or differing severity of medical conditions among participants.

The study’s findings indicated that children who were not vaccinated according to their age had longer hospital stays compared to those who were vaccinated (AHR = 1.630, 95% CI: 1.444–1.894). This study is supported by research conducted at Yekatit 12 and Bahir Dar Felege Hiwot Referral Hospitals, which showed that children who were fully or partially vaccinated for their age experienced better recovery rates than those who had not been vaccinated^14,24^. These results highlight the critical role of vaccination in reducing the length of hospital stays and enhancing recovery rates for children suffering from severe acute malnutrition.

One of the key predictors identified in this study was the presence of medical complications, which was shown to extend recovery times in cases of complicated Severe Acute Malnutrition (SAM). Specifically, the study revealed that children without medical complications recovered 33.5% faster (AHR = 1.335, 95% CI: 1.195–1.576) than those with complications. This finding is consistent with prior research from the South Wollo Amhara region and Bahirdar Felege Hiwot Hospital, which also reported longer recovery times for children with complications compared to those without^15,24^. Thus, Children with medical complications tend to have significantly longer recovery times than those without.

Research has shown that the presence of comorbidities such as tuberculosis and pneumonia can hinder recovery. A retrospective cohort study carried out in a stabilization center in Southern Ethiopia, along with another study at Jimma University Medical Center, indicated that children without tuberculosis infections were more likely to recover sooner. In contrast, those with tuberculosis (AHR = 1.565, 95% CI: 1.395–2.809) were found to have longer recovery times, which aligns with the findings of this study^11,31^. This evidence suggests that comorbidities associated with tuberculosis negatively affect recovery duration.

The findings of this study indicated that children with pneumonia were 2.558 times less likely to recover compared to those without pneumonia (AHR = 2.558, 95% CI: 2.405–2.769). This aligns with research conducted at various hospitals in Ethiopia, including Yekatit 12 Hospital^14,22^. These results highlight the critical need for early detection, timely treatment, and comprehensive care for children with pneumonia to reduce the adverse effects on their recovery and overall health outcomes.

According to this study, hospital-acquired infections were also found to hinder recovery in children. Those who contracted these infections experienced a 45.9% (AHR = 1.459, 95% CI: 1.315–1.669) longer recovery time compared to children without such infections. These infections, which occur during hospital stays, can further compromise the already weakened immune systems of these children^34,35^. This finding is significant as it underscores the ongoing impact of hospital-acquired infections on recovery times. The results indicate that these infections can have enduring effects on children’s health and well-being, potentially resulting in prolonged illness and an increased burden on healthcare resources.

The type of malnutrition at the time of admission significantly influences recovery time. The results of this study indicate that children with non-edematous malnutrition and marasmic kwash experience better recovery compared to those with edematous malnutrition (AHR = 1.88, 95% CI: 1.278–2.765; AHR = 2.00, 95% CI: 1.254–3.486, respectively). This finding aligns with a retrospective follow-up study conducted at Assosa General Hospital, which reported that children diagnosed with non-edematous malnutrition had 1.69 times higher chances of early recovery than those with edematous malnutrition. Similarly, children with marasmic kwashiorkor were 1.60 times more likely to recover early compared to those with edematous malnutrition^27^. These results underscore the importance of the type of malnutrition as a key predictor of recovery time.

### Limitation of the study

Our study faced limitations due to the restricted availability and quality of data. The retrospective and secondary nature of the data resulted in incomplete and potentially inaccurate information, which negatively impacted the reliability and validity of our findings. Additionally, we were unable to access certain critical variables, preventing us from considering factors such as maternal and paternal influences and specific characteristics of the healthcare facility. It is important to note that our study population may not fully represent all individuals with severe acute malnutrition (SAM), which could introduce selection bias and affect the outcomes. Another limitation of our study is that we treated death and loss to follow-up as censored events in the survival analysis rather than accounting for them as competing risks. This approach may have led to an overestimation of recovery; however, due to data constraint, a competing risk analysis was not feasible.

## Conclusions

The results of this study, which focused on children with severe acute malnutrition admitted to public hospitals in Jimma town, revealed an 81.7% recovery rate, with a median recovery time of 12 days (IQR: 8, 15). This indicates a favorable recovery rate along with an acceptable median survival time. However, the findings also highlight the existence of preventable and treatable factors that influence recovery duration and create additional costs for healthcare facilities, parents, and the overall healthcare system. Factors such as lack of vaccination, comorbidities like tuberculosis and pneumonia, the type of malnutrition, and anthropometric measurements at admission were identified as affecting recovery times. These insights emphasize the necessity for interventions aimed at addressing these predictive factors to enhance recovery rates and alleviate the financial burden on all parties involved in the care of these children.

### Recommendations

Based on the findings and outcomes of this study, the following recommendations can be made: It is crucial to prioritize the prompt management of comorbidities such as tuberculosis and pneumonia to prevent extended hospital stays and reduce the risk of hospital-acquired infections in children. Additionally, efforts should be made to promote universal vaccination coverage appropriate for each age group, along with community-based anthropometric nutritional assessments to identify cases of malnutrition at an early stage. Hospitals and healthcare facilities should focus on adhering to established guidelines and providing necessary medications and nutritional supplements to facilitate a swift and effective recovery for children. Furthermore, additional research should be conducted to investigate factors related to healthcare facilities, parents of affected children, and other unidentified elements that were not explored in this study, while considering the methodological limitations of using this study as a baseline.

## Supplementary Information

Below is the link to the electronic supplementary material.

Supplementary Material 1

## Data Availability

The datasets generated and analyzed during the present study are available from the corresponding author on reasonable request.

## Abbreviations

AHR: Adjusted hazard ratio
AIDS: Acquired immune deficiency syndrome
COVID-19: Coronavirus disease of 2019
HFA: Height for age
HIV: Human immune virus
IQR: Inter quartile range
IV: Intravenous
JUMC: Moderate acute malnutrition
MAM: Moderate acute malnutrition
MUAC: Mid upper arm circumference
NG –TUBE: Nasogastric tube
SAM: Severe acute malnutrition
TB: Tuberculosis
TFU: Therapeutic feeding unit
UNICEF: United nations children fund
WFA: Weight for age
WFH: Weight for height
WHO: World Health Organization
