# Supplementary material for: Duration of recovery from severe acute malnutrition and associated factors in children aged 6–59 months: a retrospective cohort study
Source: Sci Rep. 2025 Dec 23;16:186. doi: 10.1038/s41598-025-28931-5 (PMC12764471; doi:10.1038/s41598-025-28931-5)
Supplement: Supplementary file 1 — Supplementary Material 1 [file 41598_2025_28931_MOESM1_ESM.docx]

**Results of Duration of Recovery from Severe Acute Malnutrition and Associated Factors in Children Aged 6-59 Months.**

**Table S1:** Presentation of Admission and Comorbidity patterns of Under-Five Children Admitted to Public Hospitals in Jimma Town, From January 2020 to December 2021 Jimma, Ethiopia

| **Variables** | **Categories** | **Number** | **Percentage** |
| --- | --- | --- | --- |
| Pulse Rate | Normal | 217 | 67.2% |
|  | Tachycardia | 102 | 31.6% |
|  | Bradycardia | 4 | 1.2% |
| Respiratory Rate | Normal | 212 | 65.6% |
|  | Tachypnea | 107 | 33.1% |
|  | Bradypnea | 4 | 1.2% |
| Weight For Height | Normal | 81 | 25.1% |
|  | Moderate | 70 | 21.7% |
|  | Severe | 172 | 53.3% |
| Weight For Age | Normal | 65 | 20.1% |
|  | Moderate | 88 | 27.2% |
|  | Severe | 170 | 52.6% |
| Height For Age | Normal | 107 | 33.1% |
|  | Moderate | 127 | 39.3% |
|  | Severe | 89 | 27.6% |
| Type of Malnutrition | Non-Edematous | 201 | 62.2% |
|  | Edematous | 82 | 25.4% |
|  | Marasmic Kwash | 40 | 12.4% |
| Admission | New | 304 | 94.1% |
|  | Readmission | 19 | 5.9% |
| Edema | None | 202 | 62.5% |
|  | +1 | 11 | 3.4% |
|  | +2 | 45 | 13.9% |
|  | +3 | 65 | 20.1% |
| Consciousness | Alert | 281 | 87.0% |
|  | Lethargic | 40 | 12.4% |
|  | Comatose | 2 | 0.6% |
| MUAC Category | Normal | 57 | 17.6% |
|  | <11.5 cm | 199 | 61.6% |
|  | 11.5-12.5 cm | 67 | 20.4% |
| Dehydration | Yes | 108 | 33.4% |
|  | No | 215 | 66.6% |
| Diarrhea | Yes | 152 | 47% |
|  | No | 171 | 53% |
| Shock | Yes | 10 | 3% |
|  | No | 313 | 97% |
| Failed appetite test | Yes | 26 | 8.0% |
|  | No | 297 | 92% |
| Hospital acquired infections | Yes | 79 | 24.5% |
|  | No | 244 | 75.5% |

**Table S2:** Distribution of Treatment-Related Factors of Under-5 Children Admitted to Public Hospitals in Jimma Town, From January 2020 to December 2021 Jimma, Ethiopia

| **Variables** | **Variable Categories** | **Number** | **Percentage** |
| --- | --- | --- | --- |
| F-100 | Yes | 92 | 28.5% |
|  | No | 231 | 71.5% |
| F-75 | Yes | 269 | 83.3% |
|  | No | 54 | 16.7% |
| Plumpy nuts | Yes | 169 | 52.3% |
|  | No | 154 | 47.7% |
| Vitamin A | Yes | 44 | 13.6% |
|  | No | 279 | 86.4% |
| Folic acid | Yes | 43 | 13.3% |
|  | No | 280 | 86.7% |
| NG tube feeding | Yes | 30 | 9.3% |
|  | No | 293 | 90.7% |
| IV antibiotics | Yes | 225 | 69.7% |
|  | No | 98 | 30.3% |
| PO antibiotics | Yes | 99 | 30.7% |
|  | No | 224 | 69.3% |
| Dewormed | Yes | 31 | 9.6% |
|  | No | 292 | 90.4% |
| IV fluid | Yes | 2 | 0.6% |
|  | No | 321 | 99.4% |
| Blood transfusion | Yes | 4 | 1.2% |
|  | No | 319 | 98.8% |
| Additional supplementary medications | Yes | 47 | 14.6% |
|  | No | 276 | 85.4% |
| Diagnosing Professional | Health officer | 2 | 0.6% |
|  | Medical intern | 100 | 31.0% |
|  | General physician | 114 | 35.3% |
|  | Pediatrics Resident | 103 | 31.9% |
|  | Pediatrician | 4 | 1.2% |
| Hospital attended | Referral hospital | 232 | 71.8% |
|  | Primary hospital | 91 | 28.2% |

**The plots of Recovery Status and Checking Proportional Hazards Assumption for Under-Five Children Admitted to Public Hospitals in Jimma Town**

**Figure S1:** Recovery Status of Under-Five Children Admitted to Public Hospitals in Jimma Town, From January 2020 to December 2021 Jimma, Ethiopia.

**Figure S2:** Cox Snell residual graph for checking proportional hazard assumption under-five children admitted to public hospitals in Jimma town, Jimma, Ethiopia 2023
